# Losartan improves intestinal mucositis induced by 5-fluorouracil in mice

**DOI:** 10.1038/s41598-021-01969-x

**Published:** 2021-12-01

**Authors:** Maisie Mitchele Barbosa Oliveira, Aurigena Antunes de Araújo, Susana Barbosa Ribeiro, Polyana Crislayne Moreira de Sales Mota, Vitória Barros Marques, Conceição da Silva Martins Rebouças, Jozi Godoy Figueiredo, Patrícia Batista Barra, Gerly Anne de Castro Brito, Renata Ferreira de Carvalho Leitão, Gerlane Coelho Bernardo Guerra, Caroline Addison Carvalho Xavier de Medeiros

**Affiliations:** 1 Post Graduate Program Biotechnology-RENORBIO, Federal University of Rio Grande do Norte (UFRN), Natal, RN Brazil; 2 Post Graduate Program in Pharmaceutical Science, Post Graduate Program Dental Sciences, Department of Biophysics and Pharmacology, Federal University of Rio Grande do Norte (UFRN), Natal, RN Brazil; 3 Junior Postdoctoral Student CNPq-Federal University of Rio Grande do Norte (UFRN), Natal, RN Brazil; 4 Biosciences Center, Federal University of Rio Grande do Norte (UFRN), Natal, RN Brazil; 5 Department of Morphology, Faculty of Medicine, Federal University of Ceará (UFC), Fortaleza, CE Brazil; 6 Department of Biochemistry, Faculty of Vale do São Lourenço (EDUVALE), Jaciara, MT Brazil; 7 Post Graduate Program in Biology Teaching in National Network-PROFBIO, Department of Biomedical Sciences, State University of Rio Grande do Norte (UERN), Mossoró, RN Brazil; 8 Post Graduate Program Morphofunctional Sciences, Post Graduate Program Medical Sciences, Department of Morphology, Faculty of Medicine, Federal University of Ceará (UFC), Fortaleza, CE Brazil; 9 Post Graduate Program Morphofunctional Sciences, Department of Morphology, Faculty of Medicine, Federal University of Ceará (UFC), Fortaleza, CE Brazil; 10 Post Graduate Program Biochemistry and Molecular Biology, Post Graduate Program Pharmaceutical Science, Department of Biophysics and Pharmacology, Federal University of Rio Grande do Norte (UFRN), Natal, RN Brazil; 11 Post Graduate Program Biotechnology-RENORBIO, Post Graduate Program Biochemistry and Molecular Biology, Department of Biophysics and Pharmacology, Federal University of Rio Grande do Norte (UFRN), Natal, RN Brazil

**Keywords:** Biochemistry, Cancer, Gastroenterology, Oncology

## Abstract

Intestinal mucositis (IM) is a common side effect of 5-fluorouracil (5-FU)-based chemotherapy, which negatively impacts therapeutic outcomes and delays subsequent cycles of chemotherapy resulting in dose reductions and treatment discontinuation. In search of new pharmacological alternatives that minimize your symptoms, this work set out to study the effect of losartan (LOS), a receptor type I (AT1) angiotensin II antagonist, on intestinal mucositis induced by 5-FU. Intestinal mucositis was induced by a single intraperitoneal administration of 5-FU (450 mg/kg) in Swiss mice. Losartan (5, 25 or 50 mg/kg) or saline was orally administered 30 min before 5-FU and daily for 4 days. On 4th day, the animals were euthanized and segments of small intestine were collected to evaluate histopathological alterations (morphometric analysis), concentration of inflammatory cytokines, oxidative stress markers and genic expression of NF-κB p65, Fn-14 and TWEAK. Weight evaluation and changes in leukogram were also analyzed. 5-FU induced intense weight loss, leukopenia and reduction in villus height compared to saline group. Losartan (50 mg/kg) prevented 5-FU-induced inflammation by decreasing in the analyzed parameters compared to the 5-FU group. Our findings suggest that 50 mg/kg of losartan prevents the effects of 5-FU on intestinal mucosa in mice.

## Introduction

Intestinal mucositis (IM), a common side effect of 5-Fluorouracil (5-FU)-based anticancer regimens, is a complex process that leads to inflammatory and/or ulcerative lesions^1–4^. This inflammatory condition may limit the patient’s ability to tolerate chemotherapy, compromising cancer therapy and also contributes to higher hospitalization costs^5^.

Chemotherapy-induced IM has been linked to symptoms such as nausea, dyspepsia, dysphagia, diarrhea, loss of appetite, malnutrition and pain. Intestinal damage is microscopically characterized by shorted villi, loss of crypt architecture and inflammatory cell infiltration along the intestinal wall resulting in loss of mucosal integrity and bacterial colonization^6–9^. Its physiopathology has been described as a sequence of interrelated biological events compromising epithelial integrity and gastrointestinal dysmotility. The last phase is the recomposition of the structure of the epithelium^10,11^.

5-Fluorouracil is a pyrimidine antimetabolite, widely prescribed against gastric and colorectal cancer, which interferes with the synthesis of RNA and DNA, leading to cell apoptosis and tissue damage^12^. In addition to the direct cell damage by chemotherapy, there is the release of mediators of oxidative stress and the nuclear factor kappa B (NF-κB), the most studied signaling pathway of IM, which induces the expression of cytokines proinflammatory drugs such as interleukin 1 beta (IL-1β) and tumor necrosis factor alpha (TNF-α) that promote damage and rupture in the intestinal epithelium barrier.

Losartan, a AT1 angiotensin 2 receptor blockers (ARB), used clinically for antihypertensive purposes, has anti-inflammatory effects widely described in the literature^13–17^. Losartan has been shown to reduce pro-inflammatory cytokines, such as TNF-α, IL-1β, IL-6, and the activation of nuclear transcription factor (NF-κB)^18,19^, in addition to an antioxidant effect^13,20^ and the ability to attenuate gastric mucosal damage when used in combination with non-steroidal anti-inflammatory drugs (NSAIDs)^21^.

Despite its impact on patients, there are currently no effective treatment options to prevent or treat intestinal mucositis associated to chemotherapy^22^, so the search for new therapeutic targets is relevant. Our research group had already shown that angiotensin 2 pathway modulators have a protective effect on oral^23^ and intestinal mucositis in rats^24^. However, as far as we know, the effect of losartan on 5-fluorouracil-induced intestinal mucositis has not yet been described. Thus, the aim of the present study was to investigate the effect of losartan in the prevention of intestinal mucositis, evaluating morphological changes in the intestine and parameters related to oxidative stress and inflammation.

## Results

### Histopathological evaluation and intestinal morphometry

**Figure 1:**
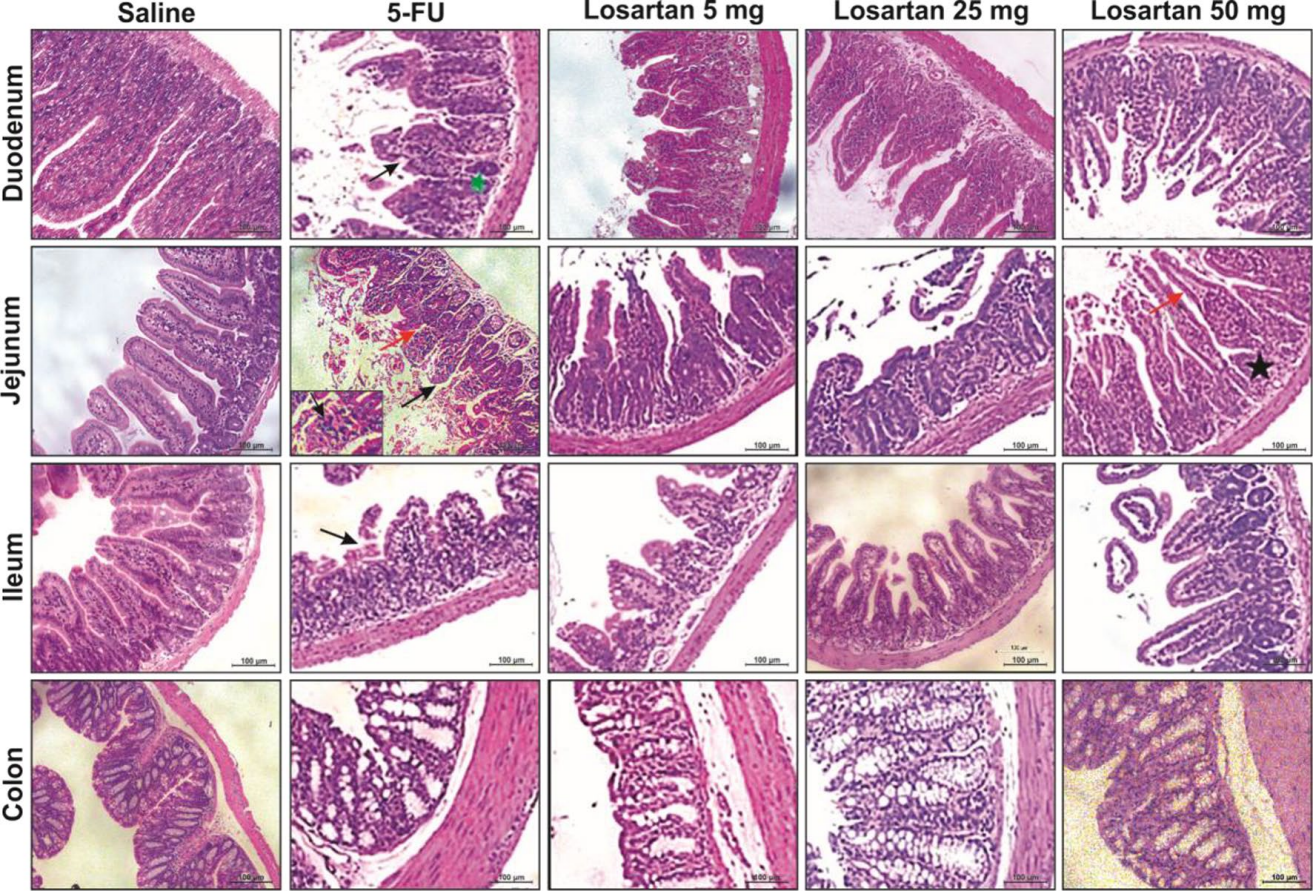
Histopathological analysis of intestinal segments of the duodenum, jejunum, ileum and colon of animals submitted to intestinal mucositis (IM) induced by 5-fluorouracil. Photomicrographs on a 100 μm scale demonstrated that the animals in the saline group maintained the integrity of the intestinal mucosa, with villi and crypts without damage, as well as the absence of inflammatory infiltrate. The 5-FU caused the loss of integrity, structure and shortening of the villi (black arrow), injury and disruption of the crypts with the presence of vacuolization (green arrow) and the presence of inflammatory infiltrate (red arrow). Losartan treatment at a dose of 50 mg/kg decreased the effects caused by 5-FU, improving the appearance of villi (red arrow) and crypts (black arrow).

**Figure 2:**
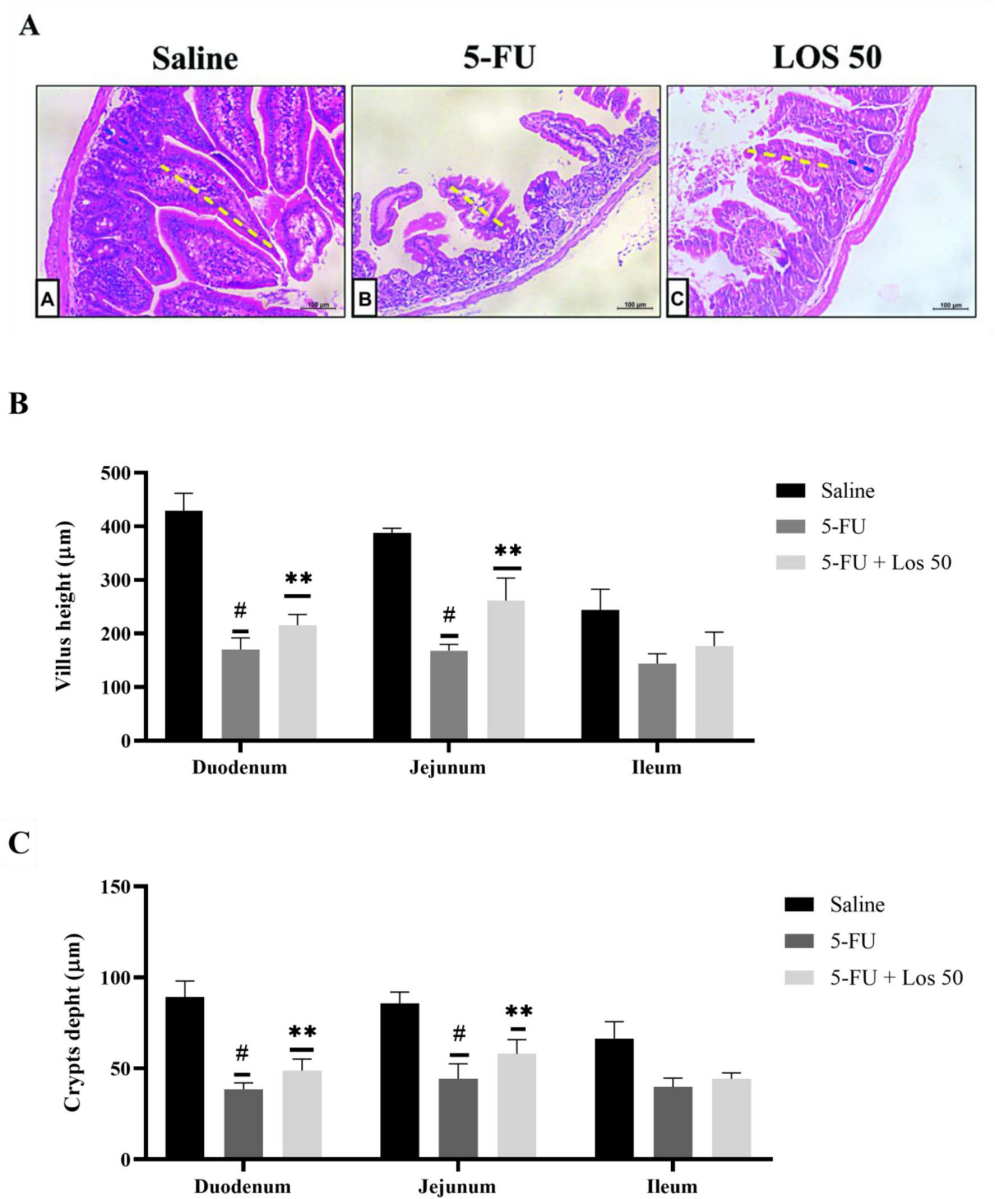
(**A**) Images of histological slides stained in H&E, of fragments of the jejunum of mice submitted to intestinal mucositis. Animals saline without IM (a), with intestinal mucositis induced by 5-FU, without treatment (b) and animals with IM, treated with losartan at a dose of 50 mg/kg. The measurement of villus integrity is represented by dashed lines in yellow and the size of crypts is represented by dashed lines in blue. Segments of the duodenum, jejunum and ileum were collected to measure the villus height (10 villi/lamina). Scale bars = 100 µm (**B**) and crypt depth measurements (**C**). The bars represent the mean ± SEM of 5 mice in each group. ^#^p < 0.001 vs. saline group, **p < 0.001 vs. 5-FU group. One way ANOVA with Bonferroni post-test.

5-FU induced intestinal damage in all intestinal segments investigated, and this damage involved shortened villi, loss of crypt architecture, vacuolization and edema in the mucosal, submucosal and muscle layer and a pronounced inflammatory cell infiltrate compared to the control group (Fig. 1). Losartan 50 mg/kg significantly prevented intestinal injury (Fig. 1). Intestinal damage was also evaluated by blind semi-quantitative analysis (Table 1). All intestinal segments showed moderate to severe intestinal damage in 5-FU administered mice and losartan (50 mg/kg) significantly attenuated this injury in the jejunum and colon (Table 1). The deleterious effects of 5-FU were also visible in the morphometry of small intestine (Fig. 2A), which showed a significant reduction in the villus height (Fig. 2B) and crypt depth (Fig. 2C) in the duodenum and jejunum compared to the control group. Losartan (50 mg/kg) significantly prevented intestinal injury (Fig. 2A–C).

**Table 1:** Histological scores obtained in the experimental model of MI treated with losartan.

Segments	Experimental groups				
	Saline	5-FU	LOS 5	LOS 25	LOS 50
Duodenum	0 (0–0)	2 (2–3)*	1 (1–3)	1 (1–2)	2 (1–2)
Jejunum	0 (0–0)	3 (3–3)*	2 (2–3)	2 (1–2)	1 (1–2)**
Ileum	0 (0–0)	3 (2–3)*	3 (1–3)	2 (1–2)	2 (1–2)
Colon	0 (0–0)	3 (2–3)*	1 (1–3)	2 (1–2)	1 (1–1)**

Histological scores obtained in the experimental model of IM treated with Losartan Score data in median with respective intervals, obtained after observation of the histological slides, where scores from 0 to 3 were attributed. The data were analyzed using the Kruskal–Wallis and Dunn's test (n = 5). *Represents p < 0.05 vs. saline group and **Indicates p < 0.05 vs. 5-FU group.

### Percentage change in body mass and total leukocyte count

**Figure 3:**
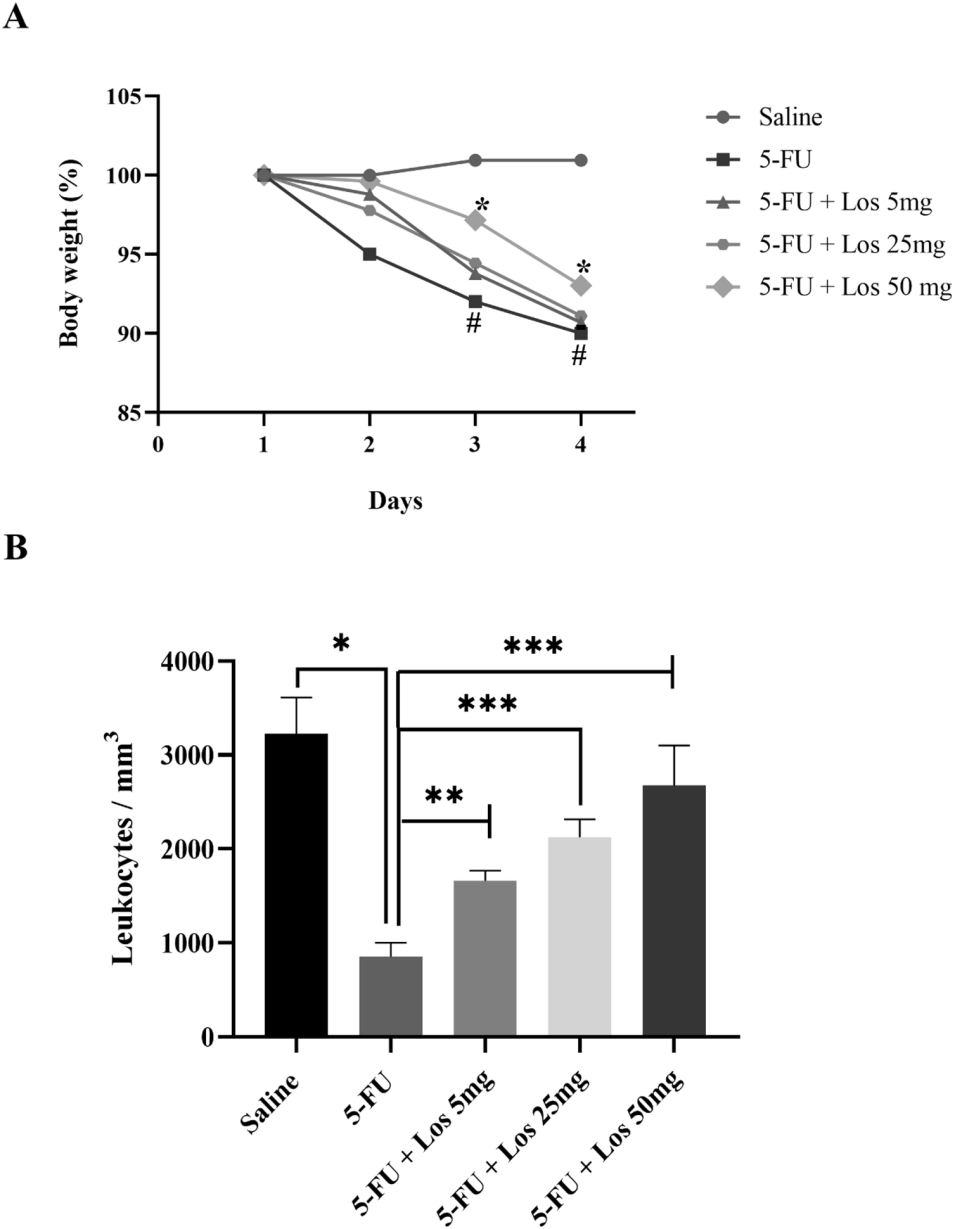
(**A**) Effect of losartan on weight loss and (**B**) total leukocyte count in mice with 5-FU-induced intestinal mucositis (IM). (**A**) Weight assessment is shown as a percentage. The bars represent the mean ± SEM (n = 5) in each group. ^#^p < 0.01 versus Saline group; *p < 0.001 versus group 5-FU (Two-way ANOVA analysis of variance followed by the Tukey test). (**B**) Animals with IM, group 5-FU, reduced the number of leukocytes compared to the saline group (**p < 0.0001). LOS 50 mg/kg significantly increased the number of cells when compared to the 5-FU group. *p < 0.0001 vs. group 5-FU; **p < 0.001 versus 5-FU group and ***p < 0.05 versus 5-FU group. ANOVA one-way test followed by the Tukey test.

5-FU caused a pronounced loss of body weight compared to the saline control group (Fig. 3A), which was partially rescued by losartan (50 mg/kg).

5-Fluorouracil significantly decreased the number of leukocytes in the circulating blood compared to the saline group (animals not submitted to IM) (Fig. 3B). This effect was prevented by the three different evaluated doses of losartan (Fig. 3B).

### Cytokine assay (IL-1β and TNF-α)

**Figure 4:**
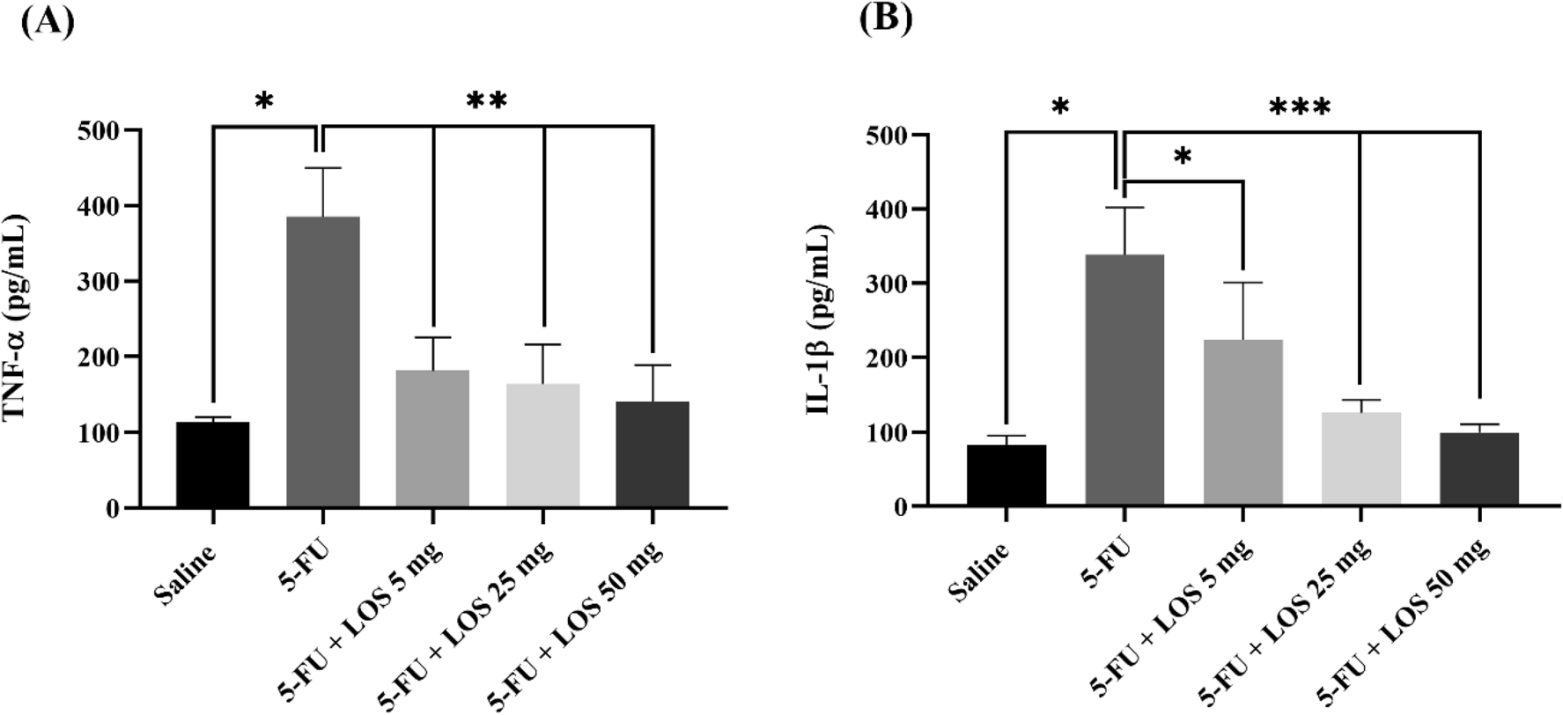
Cytokines tumor necrosis factor alpha (TNF-α) (**A**) and interleukin 1 beta (IL-1β) (**B**) in the jejunal portion of mice submitted to the experimental model of intestinal mucositis (IM). The saline group comprised animals without IM; In the 5-FU group, the animals received 5-FU and without treatment. The LOS groups received 5-FU, underwent IM and were treated with losartan (orally), in doses (5, 25 or 50 mg/kg) (n = 5/group). The results are presented as mean ± standard error of the mean (n = 5/group). *p < 0.001 (compared to saline); **p < 0.0001 (compared to 5-FU) (ANOVA analysis of variance with the Tukey post-test).

Inflammatory cytokines levels were markedly increased in the jejunum of 5-FU group compared to saline group (Fig. 4A,B). Losartan, at all doses evaluated, significantly prevented the increase in TNF-α levels (Fig. 4A) and IL-1β levels in the jejunal tissue (Fig. 4B) caused by 5-FU-induced intestinal mucositis.

### Malondialdehyde (MDA) and glutathione (GSH) assays

**Figure 5:**
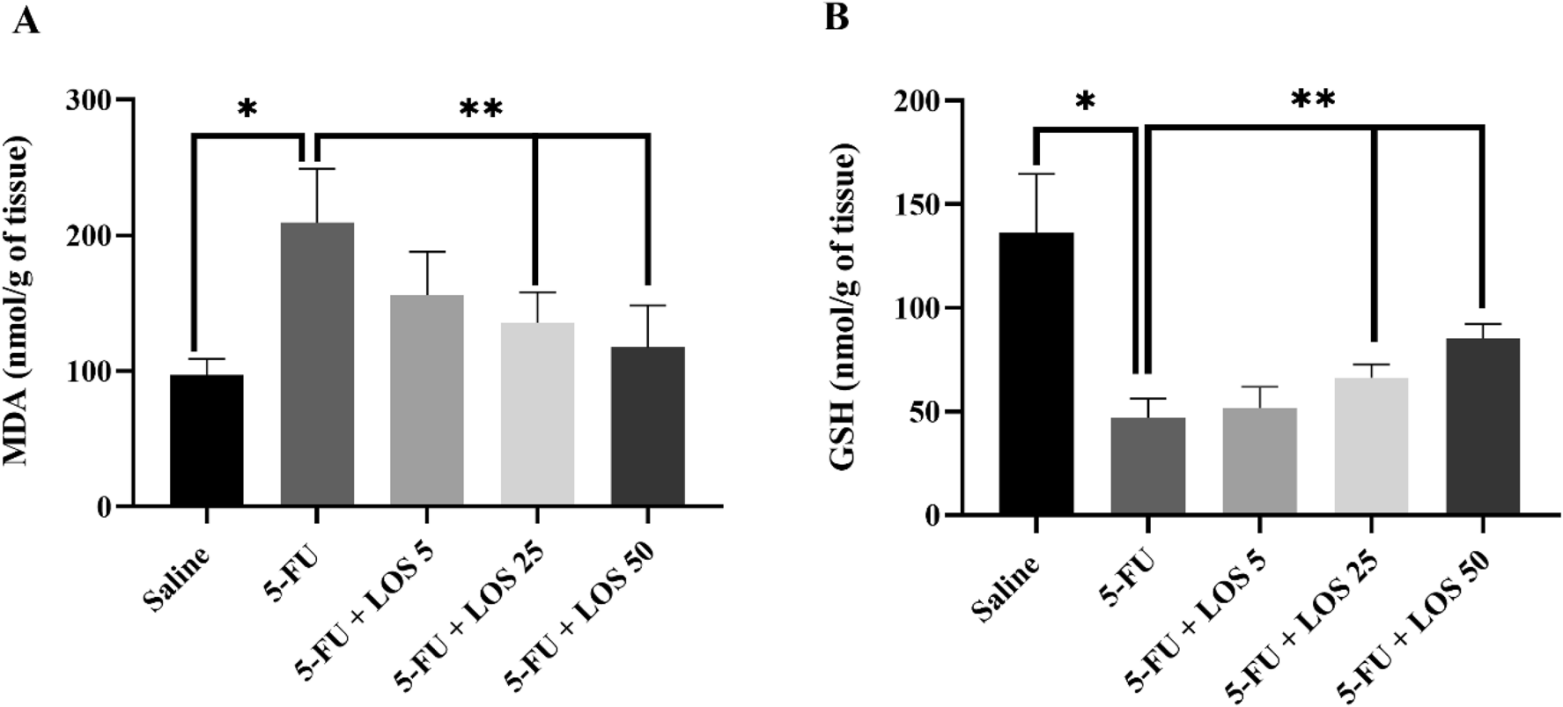
Malondialdehyde (MDA) (**A**) and glutathione (GSH) (**B**) levels in the IM model. The experimental groups saline, 5-FU and losartan are presented in doses of 5, 25 or 50 mg/kg, the values are shown as mean ± SEM. For statistical analysis, ANOVA was used, followed by the Tukey test. *p < 0.01 vs. saline group and **p < 0.01 vs. group 5-FU.

5-FU caused a significant increase in MDA levels (Fig. 5A) associated with a significant reduction in glutathione (GSH) levels (Fig. 5B) in the jejunal tissue, when compared to the saline group (p < 0.05). These effects were prevented by all doses of losartan (Fig. 5A,B).

### Reverse transcription polymerase chain reaction (RT-PCR)

**Figure 6:**
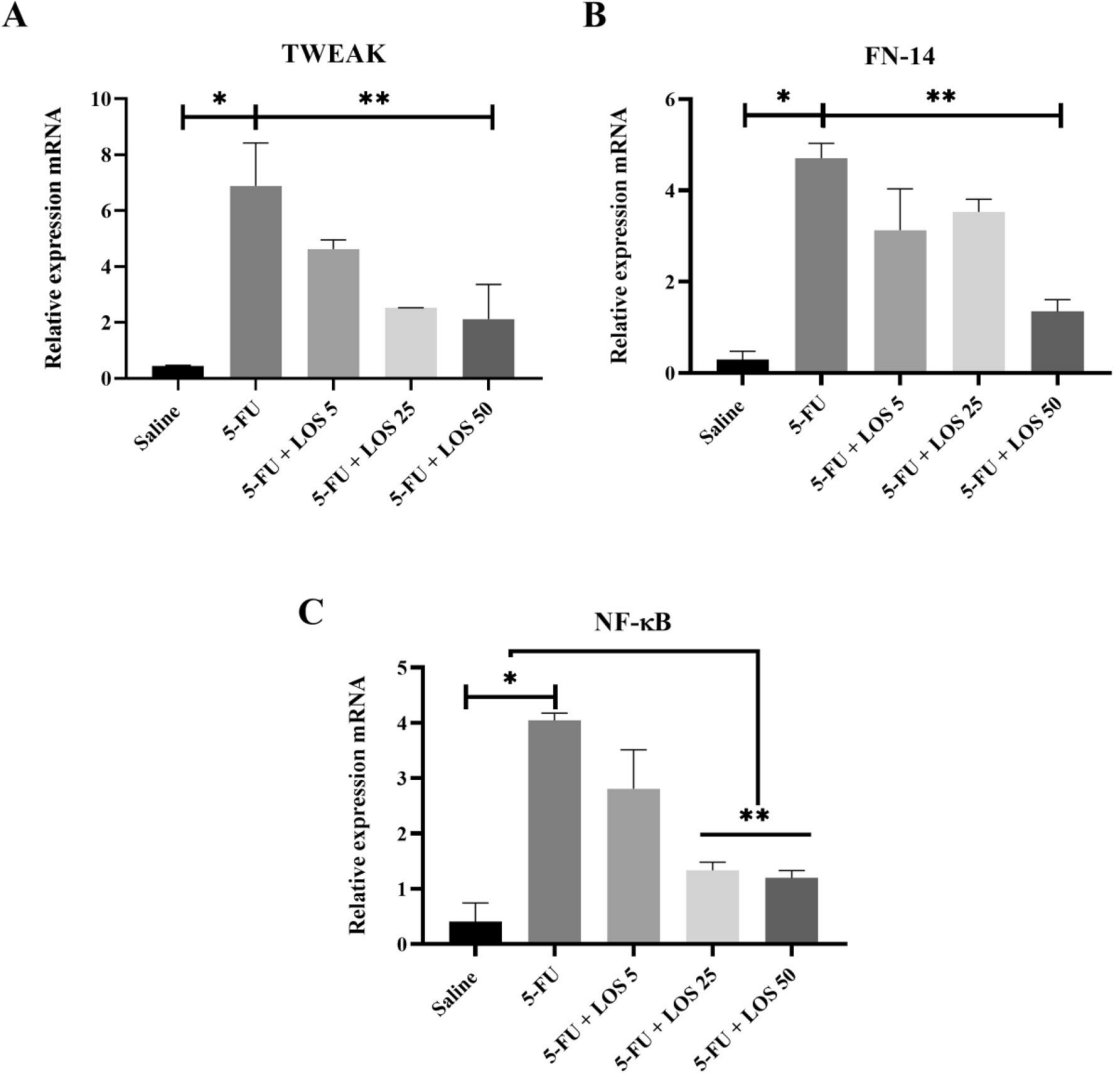
Polymerase chain reaction in real time for TWEAK, Weak apoptosis inducer like cytokine tumor necrosis factor (**A**); FN-14, inducible fibroblast growth factor 14 (**B**); NF-κB p65, the nuclear transcription factor kappa B (**C**). 5-Fluorouracil (5-FU) increased the expression of these genes compared to the Saline group. Losartan decreased the expression of TWEAK, FN-14 and NF-κB p65, compared to the 5-FU group (n = 5); *p < 0.05 compared with the Saline group; **p < 0.05 compared with the group 5-FU; analysis of variance with Tukey's post-test).

The 5-FU increased the mRNA expression of TWEAK, Fn14 and NF-κB p65, compared to the Saline group (Fig. 6). Losartan prevented the mRNA genic expression of these three markers, mainly at doses of 25 or 50 mg/kg, compared to animals with IM, without treatment (5-FU) (p < 0.05).

## Discussion

We found that 5-FU induced intestinal damage in all intestinal segments investigated, and this damage involved shortened villi, loss of crypt architecture and a pronounced inflammatory cell infiltrate in the lamina propria, associated with weight loss compared to the control group. The body weight loss might be related to malabsorption due to intestinal lesions, resulting in loss of the intestinal absorptive surface. We first investigated whether losartan affected 5-FU-induced weight loss and histological alterations. The highest dose of losartan significantly attenuated this injury in the jejunum and colon and partially rescued the pronounced loss of body weight caused by 5-FU.

According to the literature, 5-FU causes apoptosis of intestinal epithelial cells, leading to structural and inflammatory changes, well described characteristic of mucositis^25,26^. Bacterial colonization at the mucosal ulcers further induces inflammation by stimulating infiltration and activation of proinflammatory macrophages, leading to a vicious cycle of inflammation in the gastrointestinal tract^27^. Intestinal damage interferes with patient’s quality of life and diet, leading to weight loss and may compromise the delivery of cancer therapy and the overall prognosis^28–30^.

Losartan (LOS) at a dose of 50 mg/kg prevented the histopathological and morphometric changes induced by 5-FU in the jejunum and colon. In addition, LOS 50 mg/kg partially rescued the animals' weight loss induced by 5-FU-induced intestinal mucositis. Losartan is an angiotensin II AT1 receptor blocker. Although the main role of the renin-angiotensin system (RAAS) in the cardiovascular and renal systems, it has been described different subtypes of angiotensin receptors in the mucosal and muscular layers of the intestine, regulating intestinal function^31–33^. SARS plays a role in inflammation, specifically due to the action of angiotensin II. Thus, blocking the AT1 receptor promotes anti-inflammatory action^34,35^. Corroborating our findings, authors demonstrated the protective effect of losartan in inflammatory bowel diseases^36–38^.

In this investigation, 5-FU reduced the total number of leukocytes in the circulating blood of the animals, compared to the saline group. Leukopenia is an expected effect of chemotherapeutic agents and has been described in previous work on experimental mucositis^25,39,40^. The three evaluated doses of LOS prevented leukopenia, compared to the 5-FU group. This data is crucial, since leukocytes represent defense cells and can protect mice against bacterial and fungal infections, commonly associated with mucositis^41^. We believe that the effect of losartan in preventing 5-FU-induced leucopenia is not associate with its antiinflammatory effect. A recent study^42^ has shown that losartan (0.71 mg/kg, oral treatments, once a day for 50 days) caused significant increments in total white blood cells, neutrophil and monocyte values, in male rats (not treated with any chemotherapeutic agent). This effect, which probably indicates an enhancement on ability of the body to attack and destroy invading bacteria, viruses and other injurious agents, may be partially associated with its positive effect by preventing the rapid colonization of ulcers. The experimental model used in the present study does not allow us to assess whether losartan has a negative impact on 5-FU efficacy. A recent study, however, using a xenograft model of colon cancer, demonstrated that the combination of Losartan and 5-FU revealed synergistic and additive anti-tumorigenic properties^43^.

In this study, losartan reduced the levels of the pro-inflammatory cytokine’s TNF-α and IL-1β. These results are in accordance with previous data in the literature, where losartan decreased the levels of IL-1β and TNF-α in an animal model of arthritis^44^ and suppressed serum levels of pro-inflammatory cytokines such as IL-1β, IL-6, TNF-α and MCP-1 in perivasculitis, attenuating coronary inflammation in a murine model of Kawasaki’s disease^45^.

The literature have demonstrated that the role of LOS in decreasing local production of cytokines in animal models is due to the regulation of the nuclear transcription factor kappa B (NF-κB) and correlated genes, which act in the modulation of important pro-inflammatory cytokines and oxidative stress^44^. In the present study, 5-FU increased the gene expression of NF-κB p65 and LOS prevented this effect. It was observed a decreased expression of NF-κB p65 in the LOS group when compared to untreated group (5-FU group). NF-κB represents one of the most important signaling pathways in the pathophysiology of mucositis (5, 41). Chemotherapy with 5-FU promotes direct apoptosis in the basal epithelial cells, contributing to NF-κB activation, resulting in the subsequent production of the pro-inflammatory cytokines TNF-α and IL-1β. TNF-α interacts synergistically with NF-κB, amplifying its activation, also contributing to the increase in the expression of other pro-inflammatory factors, which act in tissue damage^29,30^.

**Figure 7:**
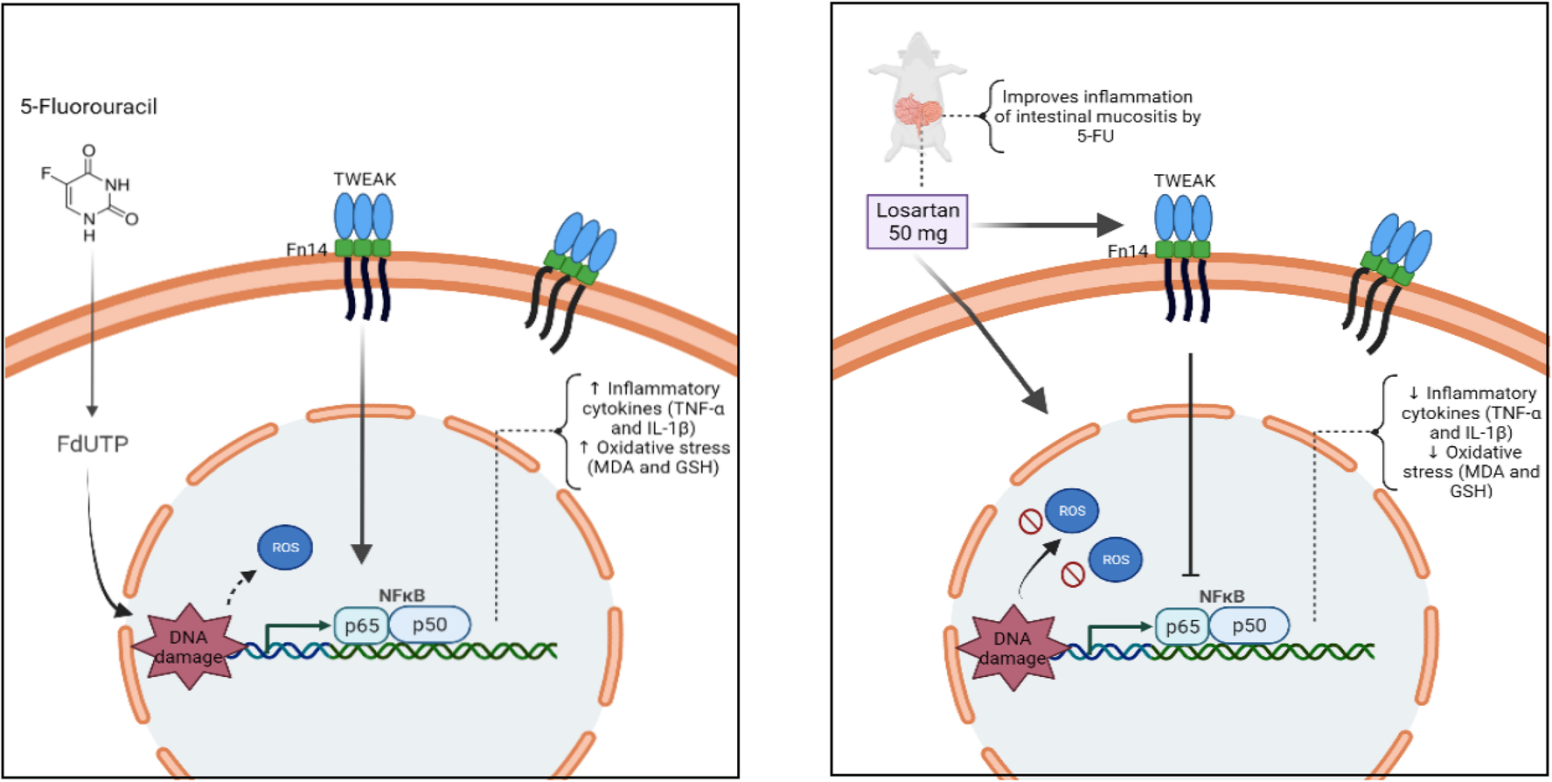
Representation of pharmacological modulation of the intestinal mucositis induced by 5-fluorouracil (5-FU) treated with the losartan. DNA damage due to 5-FU promotes the release of reactive oxygen species (ROS) and activates NF-κB that induces the expression of proinflammatory cytokines such as IL-1β and TNF-α, in addition, occurs the oxidative stress, which that together promote inflammation in the intestinal mucosa. Losartan was reducing NF-κB p65 in response the decreased of gene expression TWEAK/Fn14, reducing IL-1β and TNF-α and decreasing oxidative stress, therefore improving intestinal mucositis. TWEAK, Tumor necrosis factor (TNF)-like weak inducer of apoptosis; Fn14, Fibroblast growth factor-inducible 14; NF-κB p65, nuclear transcription factor kappa B.

In this study, LOS prevented the oxidative stress caused by lipid peroxidation, evidenced by a reduction in MDA levels and an increase in sulfhydryl groups (GSH), a classical example of a scavenging antioxidant. In other experimental models, losartan reduced inflammation and the release of free radicals, modulating antioxidant enzymes, including catalase and glutathione peroxidase^20,46^. We demonstrated that 5-FU interfered with NF-κB p65 pathway and increased gene expression of TWEAK (weak inducer of tumor necrosis factor-like apoptosis) and Fn14 (fibroblast growth factor-inducible 14), compared to the saline group. Losartan significantly prevented the increase of TWEAK and Fn14 gene expressions, induced by 5-FU. TWEAK is a cytokine of the TNF family that signals by the Fn14 receptor, participating in biological activities, including proliferation, migration, differentiation, apoptosis, angiogenesis and inflammation^47^. Angiotensin II modulates the TWEAK/Fn14 pathway in response to inflammation and myocardial injury in rats^48^. Authors suggest that blocking the TWEAK/Fn14 pathway attenuates chronic intestinal inflammation and promotes epithelial repair^49–51^. Thus, we demonstrated a important effect of losartan on 5-FU-induced intestinal mucositis, preventing histopathological and morphometric changes, reducing the oxidative stress and inflammation and modulating the NF-κB p65, TWEAK/Fn14 pathway, as illustrated in Fig. 7.

## Conclusions

Losartan 50 mg/kg partially prevented the effects of 5-FU-induced intestinal mucositis in mice. The findings of this study have to be seen in light of some limitations. More research is needed to understand the immunomodulatory effects of losartan and assess the benefits and harms (side effects) of losartan for patients undergoing 5-FU chemotherapy.

## Methods

### Animals

Female Swiss mice (*Mus musculus*), weighing 25–30 g (mean age of 8 weeks) were housed in polypropylene boxes and kept in controlled conditions of temperature (24 ± 2 °C), relative humidity of the air (50 ± 5%), 12 h light/dark cycle and access to food and water ad libitum.

### Ethical statement

All experimental protocols were approved by the Federal University of Rio Grande do Norte Ethics Committee on the use of Animals (CEUA. No. 132.059/2018) and performed in accordance with the ARRIVE ethical guidelines. All methods were performed in accordance with relevant guidelines and regulations.

### Induction of experimental intestinal mucositis (MI)

Intestinal mucositis was induced by a single administration of 5-fluorouracil (5-FU; 450 mg/kg; i.p.), as previously described^9^. On day 4, the mice were euthanized with an overdose of thiopental (90 mg/kg, i.p.).

### Experimental groups

After two weeks of acclimation to the laboratory environment. Thirty five *swiss* mice were divided into 5 groups (n = 7 per group): *saline* (healthy mice, not submitted to intestinal mucositis, that received a single intraperitoneal administration of 0.9% saline solution on the first day and oral administrations of saline, starting 30 min before the saline intraperitoneal injection, and daily, once a day, until euthanasia), *5-FU* (group submitted to intestinal mucositis by a single intraperitoneal administration of 5-fluorouracil (450 mg/kg) and received oral administrations of saline, starting 30 min before the 5-FU injection, and daily, once a day, until euthanasia; *Losartan*—LOS 5, 25 or 50 (animals submitted to 5-FU-induced intestinal mucositis and treated with oral administrations of 5, 25 or 50 mg/kg of losartan, starting 30 min before 5-FU administration and daily, once a day, until euthanasia.

Tablets of losartan were macerated using a pestle and a porcelain mortar and dissolved in of saline to obtain a concentrated solution from which the different concentrations of losartan evaluated in the present study were prepared.

### Histopathological analysis

After euthanasia, on day 4, the entire intestine was removed, washed with 0.9% saline and divided into segments (duodenum, jejunum, ileum and colon). A portion of each segment was fixed in 10% buffered formaldehyde, dehydrated and embedded in paraffin. Sections (5 µm thick) were obtained for hematoxylin and eosin (HE) staining and *subsequent* evaluation *using light microscope* (Olympus BH-2) at 200 × magnification, by an experienced observer, in a blinded manner. The severity of mucositis was graded using a modification of Leitão et al. (2011) histopathological grading system previously described^52^, considering microscopic findings such as villus and crypt integrity, inflammatory cell influx, vacuolization and edema in the mucous layer and submucosal layer (Table 2).

**Table 2:** Histopathological grading scores.

Scores	Description of the findings
0	Histological findings are normal
1	Loss of crypt architecture and villus shortening, sparse inflammatory cell infiltration, presence of vacuolization and edema in the mucous layer and normal muscle layer
2	Villus blunting with flattened and vacuolated cells, crypt necrosis, moderate inflammatory cell infiltration, vacuolization and edema in the mucosal and submucosal layers and normal muscle layer
3	Villus blunting with flattened and vacuolated cells, crypt necrosis, intense inflammatory cell infiltration, vacuolization and edema in the mucosal and submucosal layers and muscle layer showing edema, vacuolization and neutrophilic infiltration

Source: Adapted from Leitão et al. (2011).

### Intestinal morphometry

For the morphometric analyses, the length of intestinal villi, depth of the Lieberkuhn crypts and villus/crypt ratios were determined, as previously described^53^ from HE slides using a light microscope equipped with a high-resolution digital camera (LEICA^®^), connected to the computer with an image capture program. Between 5 and 10 villi and crypts were measured per slice (seven samples for each group). All morphometric measurements were performed blindly with the ImageJ^®^ software (NIH, Bethesda, MD, USA). The conversion of the measurements obtained, of villi and crypts, into pixels were converted into micrometers (μm), using the ratio 100 μm.

### Percentage change in body mass and total leukocyte count

The animals were weighed daily until the 4th day of the experimental protocol and the variation in body mass was determined, as previously described. The values obtained were expressed as a weight variable in relation to the initial weight^54^.

Immediately before euthanasia, blood samples were collected from the heart after anesthesia with an overdose of thiopental (90 mg/kg, i.p.), and diluted (1:20) in Turk’s solution (380 μL). The leukocyte count per mm^3^ of blood was determined using a Neubauer chamber. The results were expressed in number of leukocytes per mm^3^ in blood^55^.

### Cytokine assay (IL-1β and TNF-α)

Jejunum samples removed on day 4 were stored at − 80 °C until required for analysis of cytokines. The samples were homogenized and processed as previously described^29^. The concentrations of IL-1β and TNF-α in the samples were determined using a commercial ELISA kit (R&D Systems, Minneapolis, MN, USA). Briefly, microtiter plates were coated overnight at 4 °C with antibodies against IL-1β (detection range: 62.5–4000 pg/mL; sensibility or lower limit of detection: 12.5 ng/mL of recombinant mouse IL-1β) and TNF-α (detection range: 62.5–4000 pg/mL; sensibility or lower limit of detection: 50 ng/mL of recombinant mouse TNF-α). After blocking the plates, the samples and standard at various dilutions were added in duplicate and incubate at 4 °C for 24 h. After washing the plates (three times with buffer) biotinylated polyclonal anti-IL-1β or anti-TNF-α, diluted 1:1000 with assay buffer 1% BSA, was added to the wells. After further incubation at room temperature for 1 h, the plates were washed and streptavidin-HRP, diluted 1:5000, was added to each well. The chromogenic reagent O-phenylenediamine was added 15 min later and the plates were incubated in the dark for 15 min. The enzymatic reaction was interrupted with H_2_SO_4_ and the absorbance was measured at 490 nm using UV–VIS spectrophotometry. The results are expressed as pg/mL^56^.

### Malondialdehyde (MDA) and glutathione (GSH) assays

Lipid peroxidation was measured by the indirect method of quantification of malondialdehyde, based on the reaction with the chromogenic agent 1-methyl-2-phenylindole. Briefly, the jejunum tissues (5 per group) were homogenized in Tris–HCl buffer 1:5 (weight/volume) and, subsequently, centrifuged at 4 °C, in 2500*g* for 10 min. Finally, the samples were submitted to the spectrophotometer, using an absorbance of 586 nm for reading. The results are expressed in nanomoles of MDA per gram of intestinal tissue^57^.

Jejunum tissue samples were collected on day 4 and homogenized in 1 mL of 0.02 M EDTA to measure glutathione (GSH) levels, as previously described^58^. Then, supernatants (400 µL) were removed and added in 320 µL of distilled water, plus 80 µL of trichloroacetic acid (50%, w/v). The eppendorfs were centrifuged at 3000 rpm for 15 min and supernatants were transferred (100 µL) and mixed with 200 µL of Tris buffer (0.4 M, pH 8.9), followed by the addition of DTNB (5,5-dithiobis (2-nitrobenzoic acid) (0.01 M). The absorbance of GSH was read at 412 nm and the concentration was expressed in pg/mL of tissue^59^.

### Reverse transcription polymerase chain reaction (RT-PCR)

A portion of the jejunum was stored in eppendorf vials at − 80 °C for further analysis of biological and molecular markers.

The RNA was extracted from jejunum samples (n = 5) collected on day 4 using TRIzol™ reagent (Invitrogen Co., USA) and the Total RNA Isolation System S.V. (Promega, Madison, WI), resulting in the cDNA. For synthesis of the first strand cDNA, the total RNA kit was used for reaction with the ImProm-IITM reverse transcriptase system for RT-PCR (Promega) according to the manufacturer's protocol. Quantitative real-time PCR analyzes of NF-κB p65, Fn-14 and TWEAK (Table 3) mRNAs were performed using SYBR Green Mix in the Applied Biosystems^®^ 7500 FAST system (Applied Biosystems, Foster City, CA), according to a standard protocol for use with Primers (Table 3). The expression data were standardized using the reference gene *Beta* (*β*)*-actin* in the formula 2^−ΔΔCt^^60^.

**Table 3:** Primer sequences used to detect target genes in the experiment qRT-PCR.

Gene	Species	Sequences
ACTB *Beta* (*β*)*-actin*	*Mus musculus*	5′ → 3′ = AGGCCAACCTGTAAAAGATG
		3′ → 5′ = TGTGGTACGAGAGGCATAC
NFκB p65 *Nuclear factor kappa B* (*p65*)	*Mus musculus*	5′ → 3′ = CCGTCTGTCTGCTCTCTCT
		3′ → 5′ = CGTAGGGATCATCGTCTGCC
Fn-14 *Fibroblast growth factor-inducible 14*	*Mus musculus*	5′ → 3′ = AGGCTACTGTGGCCCATTCTG
		3′ → 5′ = CCCTCTCCACCAGTCTCCTCTA
TWEAK *Tumor necrosis factor* (*TNF*)*-like weak inducer of apoptosis*	*Mus musculus*	5′ → 3′ = TGCCTTGGCCTCCTGCTGGTCGT
		3′ → 5′ = GCCGGACTAGTTGTTCCAAGAAA

## Abbreviations

5-FU: 5-Fluorouracil
Fn-14: Fibroblast growth factor-inducible 14
GSH: Glutathione
HE: Hematoxylin and eosin
IL-1β: Interleukin 1 beta
IM: Intestinal mucositis
LOS: Losartan
MDA: Malondialdehyde
NF-κB: Nuclear transcription factor
NSAIDs: Non-steroidal anti-inflammatory drugs
TNF-α: Tumor necrosis factor alpha
TWEAK: Tumor necrosis factor (TNF)-like weak inducer of apoptosis
